# Case Report: *Tropheryma whipplei* Hide in an AIDS Patient With *Pneumocystis* Pneumonia

**DOI:** 10.3389/fpubh.2021.663093

**Published:** 2021-08-13

**Authors:** Jun Yan, Binhai Zhang, Zhongdong Zhang, Jinchuan Shi, Shourong Liu, Jianjiang Qi, Mengyan Wang

**Affiliations:** Department II of Infectious Diseases, Xixi Hospital of Hangzhou, Zhejiang University, Hangzhou, China

**Keywords:** *Tropheryma whipplei*, *Pneumocystis* pneumonia, HIV, case report, NGS

## Abstract

**Introduction:***Pneumocystis* pneumonia (PCP) is one of the most common opportunistic infections in HIV-infected patients. However, coinfection with *Tropheryma whipplei* is infrequent in AIDS patients with PCP.

**Case Presentation:** We report a 28-year-old male AIDS patient coinfected with *T. whipplei* and *Pneumocystis jirovecii* diagnosed in the bronchoalveolar lavage. After sulfamethoxazole–trimethoprim and meropenem treatment, the patient showed clinical improvement in 2 weeks.

**Conclusion:** Clinicians need to be alert to the occurrence of *T. whipplei* infection in AIDS patients with PCP and timely diagnosis and antibacterial treatments are essential. This case may help clinicians for timely diagnosis of the coinfection of *T. whipplei* and *P. jirovecii* in AIDS patients.

## Introduction

*Pneumocystis* pneumonia (PCP) is one of the most common opportunistic infections in HIV-infected patients with high morbidity and mortality. The in-hospital mortality rate has been decreasing since the implementation of combination antiretroviral therapies and chemoprophylaxis. However, the reported mortality rate for PCP still ranged from 12.8 to 33.1% after antiretroviral therapies and chemoprophylaxis (1, 2). *Tropheryma whipplei* pneumonia is very infrequent and easy to be overlooked, especially in AIDS-PCP patients. *T. whipplei* can cause endocarditis, encephalitis, and other acute infections. *T. whipplei* was first detected in Whipple disease which was considered as a metabolic disease in the 20th century (3). In this study, we first report a case of *T. whipplei* coinfection with *Pneumocystis jirovecii* in an AIDS patient.

## Case Presentation

**Figure 1:**
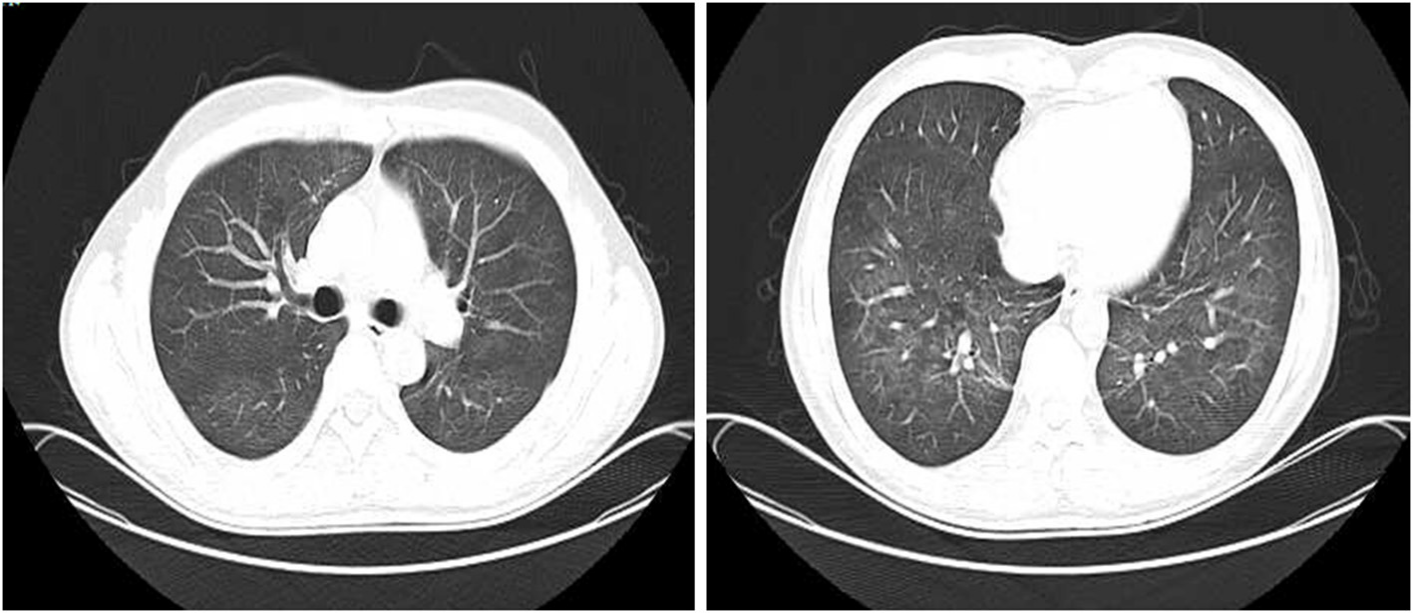
CT of the lung revealed multifocal bilateral ground-glass opacities on admission.

A 28-year-old male patient had a dry cough, chest tightness after exercise, and shortness of breath for 1 week. He took oral cephalosporin for 3 days without any efficiency, and his symptoms got worse. On admission, leukoplakia in the mouth, little wet rales at the bottom of both lungs, and multiple swollen lymph nodes were found on clinical examination. His temperature was 37.6°C. His blood test results showed that the white blood cell count was 6.52^*^10^9/*L*^, with an elevation in neutrophil ratio of 72.7%, high level of C-reactive protein (CRP) at 166 mg/L and serum amyloid protein A at 130 mg/L, low level of oxygen partial pressure at 87.5 mmHg and albumin at 30.3 g/L. Blood culture, antibody tests of *Legionella pneumophila, Streptococcus pneumoniae*, and *Mycoplasma pneumoniae*, and PCR-based detection of SARS-CoV-2 were negative. Cryptococcal capsular antigen, 1-3-β-D glucan detection, aspergillus galactomannan detection, and interferon-gamma release assays were negative. The viral load of HIV-RNA was 84,100 IU/ml. CD4^+^ T-cell count was 11 cells/μl. CT of the lung revealed multifocal bilateral ground-glass opacities (Figure 1). According to the clinical diagnosis of PCP, the patient was treated with four pills of sulfamethoxazole–trimethoprim (sulfamethoxazole 1.2 g, trimethoprim 240 mg) three times a day.

After treatment for 2 days (on day 3), the symptoms of the patient were more severe, and his temperature got to 39.9°C. We performed a bronchoscopy on the patient, and it showed bronchial mucosa hyperemia and bronchial inflammatory changes. Nucleated cell counts of bronchoalveolar lavage (BAL) were 20 ^*^ 10^6/*L*^. Cryptococcal antigen, 1-3-β-D glucan detection, and culture of BAL were negative. Hexamine silver staining of *P. jiroveci* and PCR-based detection of cytomegalovirus and *Mycobacterium tuberculosis* were also negative. In addition, next-generation sequencing technologies (NGS) of BAL were carried out, the BAL was sent to a laboratory, and DNA was extracted for whole genomic sequencing, purified, and sonicated to a size of 100–150 bp. The DNA libraries were constructed followed by end repair, joint connection, no bias PCR amplification, and sequenced using the MGlseq-2000 platform after quality control. High-quality sequencing data were generated by removing low-quality reads. After removing human sequences, the remaining sequencing data were aligned to the bacterial, viral, fungal, and protozoan databases.

**Figure 2:**
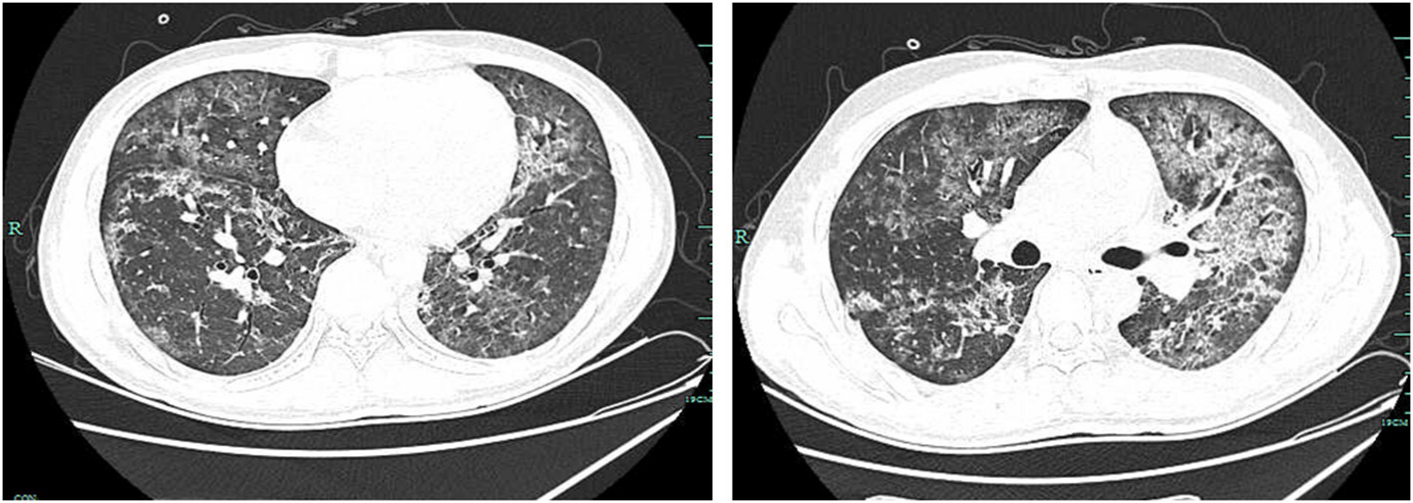
CT of the lung revealed that multiple infections occurred in bilateral lungs, and they were scattered with diffuse patchy, ground glass-like, and high density of shadows on day 6.

**Figure 3:**
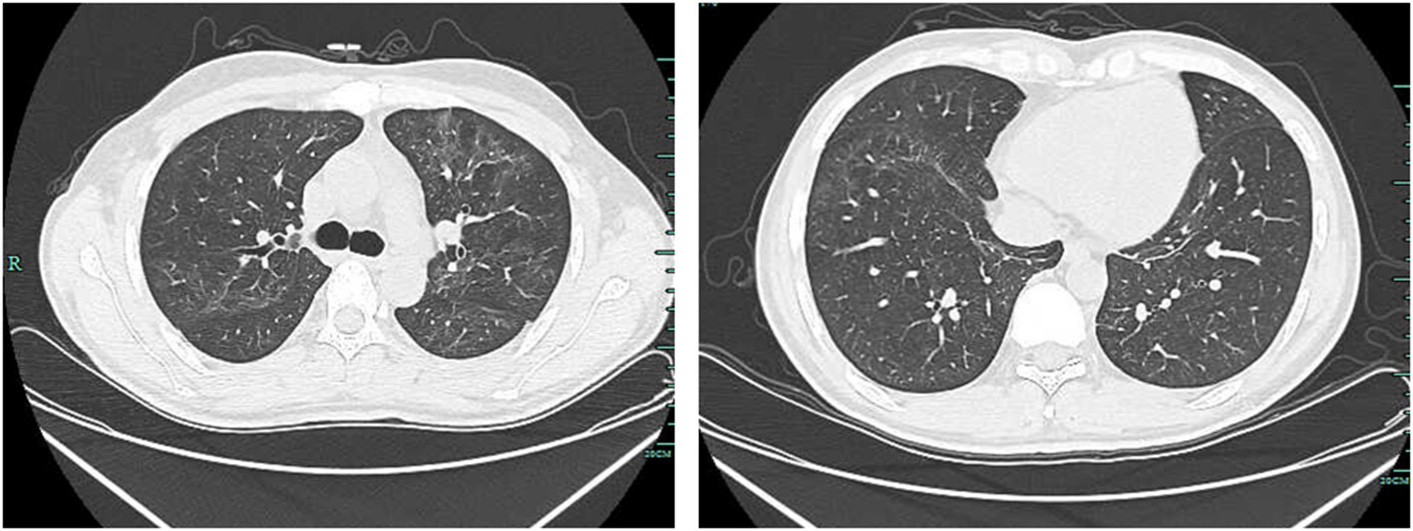
CT of the lung showed that multiple infections occurred in bilateral lungs, and they were better, slightly diffuse, and with a ground glass-like high density of shadows on day 12.

After treatment for 5 days (on day 6), CT of the lung revealed that it was worse than before (Figure 2). Results of NGS reported 205,658 unique reads of *P. jirovecii*, with coverage of identified genes 1.70% and 115 unique reads of *T. whipplei*, with coverage of identified genes 1.00% in BAL. The *P. jirovecii*-specific SYBR Green quantitative real-time PCR (qPCR) assay targeting a 301 bp fragment using a CFX96 Real-Time System was performed. The forward primer (pH207 5-ACAAATCGGACTAGGATATAGCTGGT-3) and the reverse primer pAZ102-E were used to detect the mtLSUrRNA gene (4), and the copy number of *P. jiroveci* was 4,787.36 copies/ml. For specific *T. whipplei* qPCR, the specimen was tested by using Twhi3F (5-TTGTGTATTTGGTATTAGATGAAACAG-3), Twhi3R (5-CCCTACAATATGAAACAGCCTTTG-3) primer pair, and the specific TaqMan probe Twhi3 (6-FAM-GGGATAGAGCAGGAGGTGTCTGTCTGG-TAMRA). When the specimen was positive in this assay, the result was confirmed by a second qPCR by using Twhi2F (5-TGAGGATGTATCTGTGTATGGGACA-3) andTwhi2R (5-TCCTGTTACAAGCAGTACAAAACAAA-3) primer set, and the Twhi2 probe (6-FAM-GAGAGATGGGGTGCAGGACAGGG-TAMRA) (5). The copy number of *T. whipplei* was 11,717.3 copies/ml. Considering the progressed condition of the patient, meropenem 1 g q8h and caspofungin 70 mg per day were added for treating the infections of *T. whipplei* and *P. jirovecii*. Methylprednisolone 40 mg BID was added due to the severe symptoms. On day 12, there was a clinical improvement, and CT of the lungs showed obviously better than before (Figure 3), and the patient was discharged on day 16.

## Discussion and Conclusions

A previous study had found that *T. whipplei* could easily colonize in the lungs of HIV individuals by analyzing bacterial gene sequences in BAL (6). An AIDS patient with *T. whipplei* pneumonia had been reported before, and Stein *et al*. suggested that *T. whipplei* should be considered in the differential diagnosis of pneumonia in AIDS patients (7). In this study, we reported the coinfection of *T. whipplei* and *P. jirovecii* pneumonia, which frequently had similar symptoms of fever, cough, and dyspnea (8–10), and a previous case-control study revealed that *P. jirovecii* was frequently associated with the *T. whipplei* pneumonia (11).

As we all know, lung infections by opportunistic or virulent pathogens are the major reasons that cause high mortality in AIDS patients. However, it is difficult to diagnose specific pathogens of lung infection. Due to the difficulty of culture, the diagnosis methods of *P. jirovecii* included direct cytochemical staining, immunofluorescent staining with monoclonal antibodies, and molecular methods such as PCR, with no ideal specificity and sensitivity (12). Similar to *P. jirovecii*, the culture of *T. whipplei* is difficult, which may not be suitable for the clinic. Therefore, PCR, a more specific and sensitive technique than the other methods, has become a preferred technique for diagnosing *T. whipplei* infection (13). In this report, we detected the presence of *P. jirovecii* and *T. whipplei* by NGS using BAL. NGS technology has been booming since 2014 for pathogenic testing (14). Nowadays, NGS has been widely applied to clinics.

Antibiotics including penicillin, streptomycin, tetracycline, ceftriaxone, meropenem, co-trimoxazole, doxycycline, and hydroxychloroquine have been used for the treatment of *T. whipplei* infection (13). In this case, oral sulfamethoxazole-trimethoprim treatment seems to be slow to work in the first few days. Adding intravenous meropenem for 1 week helped the patient to clinically improve.

*Pneumocystis* pneumonia is one of the common opportunistic infections in HIV-infected patients. This case highlights the occultation of acute *T. whipplei* pneumonia in an AIDS patient with PCP. Clinicians need to be alert to the occurrence of *T. whipplei* infection and timely diagnosis and antibacterial treatments are essential.

## Data Availability Statement

The raw data supporting the conclusions of this article will be made available by the authors, without undue reservation.

## Ethics Statement

The studies involving human participants were reviewed and approved by Ethics Committee of Xixi Hospital of Hangzhou. Written informed consent to participate in this study was provided by the participants' legal guardian/next of kin. Written informed consent was obtained from the individual(s), and minor(s)' legal guardian/next of kin, for the publication of any potentially identifiable images or data included in this article.

## Author Contributions

MW, JY, and BZ were involved in the clinical management of this patient. JY, JS, BZ, and ZZ collected the data of the patient. SL and JQ contributed to manuscript preparation. MW wrote the article. All authors read and approved the final manuscript.

## Conflict of Interest

The authors declare that the research was conducted in the absence of any commercial or financial relationships that could be construed as a potential conflict of interest.

## Publisher's Note

All claims expressed in this article are solely those of the authors and do not necessarily represent those of their affiliated organizations, or those of the publisher, the editors and the reviewers. Any product that may be evaluated in this article, or claim that may be made by its manufacturer, is not guaranteed or endorsed by the publisher.

## Abbreviations

AIDS: Acquired immune deficiency syndrome
PCP: Pneumocystis pneumonia
BLAF: Bronchoalveolar lavage fluid
NGS: next-generation sequencing
CRP: C-reactive protein
PCR: polymerase chain reaction.
